# Death from Cocaine Injections

**Published:** 1891-03

**Authors:** 


### Death in a Dentist's Chair from Cocaine Injections.

The *Journal fur Zahnheilkunde*, September 25, 1890, reports a case of death in a dentist's chair from injections of cocaine into the gum, given for the purpose of inducing anæsthesia for the extraction of roots of teeth. The patient was a woman twenty-nine years old, apparently very healthy, but nervous. The extraction was painless, and nothing abnormal noted. The operator withdrew from the chair to get some water for the patient to rinse her mouth with, and on his return found her motionless. Physicians were summoned and artificial respiration was practiced, but without success. The autopsy disclosed the fact that three injections had been given, which served for the extraction of three roots. The quantity of cocaine in each injection was one-third of a grain.

Dufournier reports nine cases of fatal poisoning, but none of them happened to dentists, and the *Journal* thinks the case it reports the only one occurring in the practice of a dentist. This may be true but we have heard of nearly fatal cases. The action of cocaine is so very uncertain that one must use careful judgement in its ministration. It is not safe to inject a larger quantity than one-half or three-fourths of a grain, especially into vascular tissues, because its effect is carried to the heart more rapidly and the greatest effect is produced.

---

The longest medical courses are given at the Buddhist's Lamas' University in Thibet. The student has to study for ten years. According to *Nature*, a traveler named Ptitsyn has returned from that country with a collection of medical books and drugs illustrative of the knowledge and the methods of practice in Thibet. Mr. Ptitsyn remarks that he has found over one hundred diseases described in the Buddhist literature, and of these a mythical origin is ascribed to only two. Strictly medical subjects are not studied until the fifth year of the course, the first four years being devoted to the languages and theology.

The eighth year is devoted to astrology, and philosophy is studied in the last two years.

